# Glioblastoma invasion patterns from a clinical perspective—a systematic review

**DOI:** 10.1007/s10143-024-02944-6

**Published:** 2024-11-21

**Authors:** Veronica Percuoco, Erica Herlin, Francesco Prada, Marco Riva, Federico Pessina, Victor E. Staartjes, Giuseppe Maria Della Pepa, Grazia Menna

**Affiliations:** 1 Department of Neurosurgery, University Hospital Zurich, University of Zurich, Frauenklinikstrasse 10, 8091 Zurich, Switzerland; 2 Department of Neurosurgery, Fondazione Policlinico Universitario Agostino Gemelli IRCCS, Largo A. Gemelli 8, 00168 Rome, Italy; 3 Faculty of Medicine and Surgery, University of Milan, Via Festa del Perdono, 7, 20122 Milan, Italy; 4 Focused Ultrasound Foundation, 1230 Cedars Ct Suite 206, Charlottesville, VA 22903 USA; 5 Department of Neurosurgery, IRCCS Humanitas Research Hospital, Rozzano Via Alessandro Manzoni, 56, 20089 Rozzano (MI), Italy; 6 Machine Intelligence in Clinical Neuroscience & Microsurgical Neuroanatomy (MICN) Laboratory, Department of Neurosurgery, Clinical Neuroscience Center, University Hospital Zurich, University of Zurich, Rämistrasse 100, 8091 Zürich, Switzerland

**Keywords:** Glioblastoma, Functional patterns, Prognosis, Overall survival, OS, Progression free survival, PFS

## Abstract

Glioblastoma (GBM) is the most common and aggressive primary brain tumor. Despite advances in treatment, mechanisms underlying GBM invasion remain incompletely understood. This systematic review synthesizes findings from laboratory and clinical studies to elucidate the molecular mechanisms driving GBM invasion and their implications for prognosis and therapy. This review adhered to PRISMA guidelines, conducting a comprehensive search of PubMed/Medline for studies published up to October 16, 2023. Inclusion criteria focused on studies investigating molecular mechanisms of GBM invasiveness with reported clinical outcomes (overall survival (OS) and progression-free survival (PFS). Exclusion criteria included systematic reviews, case reports, small case series, and studies limited to preclinical data. Risk of bias was assessed using the ROBINS-I tool. From 831 records, 21 studies (2198 patients) met the criteria. Key GBM invasion mechanisms included ECM degradation, vascular invasion, EMT, apoptotic regulation, cytoskeletal organization, and RNA sequencing. Vascular mechanisms were most studied. Bevacizumab resistance linked to poorer outcomes. EMT markers like TWIST and ECM degradation via MMPs such as CD147 correlated with decreased survival. Cytoskeletal and RNA studies highlighted the prognostic significance of tumor subtypes and microenvironmental interactions. This systematic review elucidates the molecular mechanisms underlying GBM invasiveness and their clinical implications. Integrating molecular profiling into routine clinical assessment may enhance prognostic accuracy and therapeutic efficacy, paving the way for personalized treatment strategies.

## Introduction

Glioblastoma (GBM) is the most common and aggressive primary brain tumour with median survival from diagnosis of 15 months [1]. The old adjective “multiforme” suggests its heterogeneous clinical presentation, histopathological classification as well as response to therapy [2]. Standard treatment is based on gross total resection (GTR) when feasible [3], followed by combined radiotherapy and chemotherapy with temozolomide (TMZ) [1]. Despite this aggressive treatment approaches, recurrence is considered almost inevitable [4, 5]. This is due to its highly infiltrative growth, which makes complete surgical removal nearly impossible, and its inherent resistance to therapies. The tumors heterogeneity and the presence of resilient cancer stem cells further complicate treatment, as these factors allow some cells to survive and drive recurrence. Additionally, the blood–brain barrier limits the effectiveness of systemic treatments. WHO 2021 classification unveiled the importance of integration of molecular data in glioma classification and prognosis [6, 7]. To date, its mechanisms of invasion are not fully understood. Several key mechanisms have been identified that contribute to the invasiveness of gliomas, which also impact prognosis. For example, alterations in the extracellular matrix (ECM), vascular invasion, and other molecular changes play significant roles. ECM provides structural and biochemical support to surrounding structure, and its degradation can significantly enhance tumour cell invasion [8]. Matrix metalloproteinases (MMPs) are gaining increasing attention in this scenario since, through the breakdown of ECM components, they not only create pathways for tumour cell migration, but also release growth factors stored in the ECM, which further promotes tumour progression [9]. This unveils crucial steps in cancer invasion and potential therapeutic targets. However, cancer biology remains a nebulous field for most neurosurgeons, and more clinical research is required to fill this gap [10–14]. This study attempts to provide neurosurgeons a concise yet systematic overview of the main molecular alterations responsible for GBM invasion and aggressiveness – besides those included/discussed in the WHO 2021 classification—such as ECM matrix alterations, vascular invasion and its correlation with prognosis, survival, and therapeutic potential. Therefore, the goal of the present systematic review is to identify the molecular mechanisms underlying invasiveness and aggressiveness in high-grade gliomas that are recognized as therapeutic targets and have a significant impact on prognosis.

## Materials and methods

This systematic review followed established guidelines (e.g., PRISMA) to identify and critically appraise relevant studies [10, 11]. All steps were carried out in accordance with the Cochrane Handbook of Systematic Reviews and Meta-analysis of Interventions (version 6.3) [12]. One Electronic database (PubMed/Medline) was searched using comprehensive search terms incorporating MeSH headings and keywords related to “(glioblastoma) AND (functional) AND (patterns of invasion)” in different combinations such as "(Invasiveness) AND (aggressiveness) AND (functional patterns)”. The latest research was conducted on the 16th of October 2023. Any discordance was solved by consensus with a third, senior author.

Exclusion criteria were as follows: systematic reviews, case reports, small case series with less than 6 patients, studies reporting exclusively pre-clinical data, and studies not reporting information on clinical outcomes (OS/PFS). Other than GBM tumor types were also excluded. Non-English literature was excluded, and those studies whose full text was not retrievable were also not included. GBM definition has evolved over the years, and the term "high-grade glioma" now encompasses both astrocytoma IDH-mutated grade IV and high-grade glioma IDH wild type. Since most studies were published before this classification change, we used the most updated definition relative to the year of publication.

A systematic abstract screening of the references (forward search) was performed to identify additional records. Extracted data were categorized based on the main molecular mechanisms studied. For each paper, the following data were extracted: study design, year of publication, number of patients, mean patient age and sex distribution, as well as at least one of the two outcomes of interest (OS and PFS).

Risk of bias was assessed using the ROBINS-I tool for non-randomized studies and interventions [13]. The risk of bias was inserted for each study on the ROBINS-E tool [14], and a graph was created using the Robvis (visualization tool) [15].

## Results

**Fig. 1:**
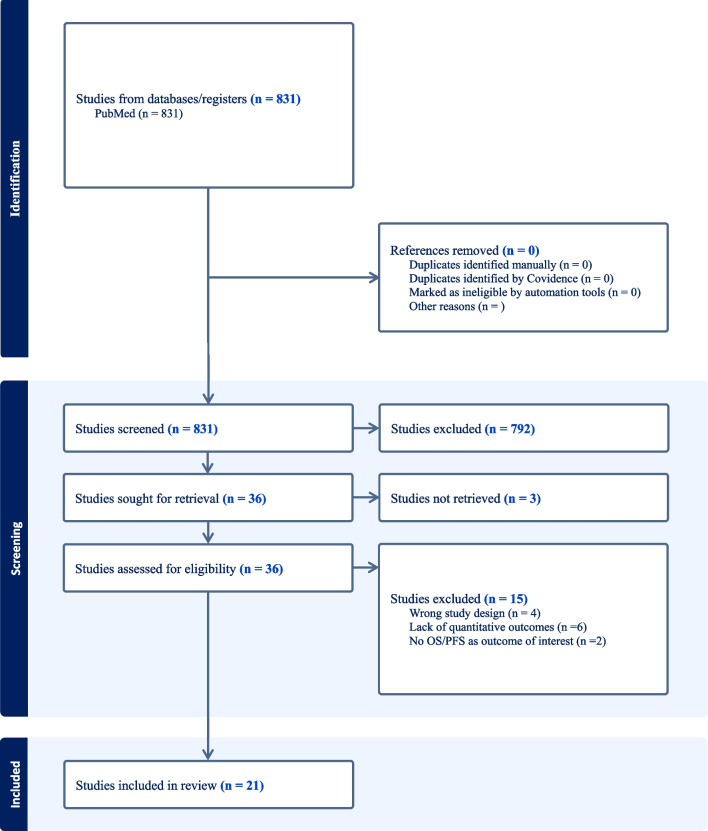
PRISMA Flowchart showing the methodological framework

The search of the literature yielded a total of 831 results. All papers were screened, and 792 records were excluded via title and abstract screening; 36 studies were found to be relevant to our research question and were assessed for eligibility (Fig. 1). Upon full-text review, 3 studies were excluded due to missed full text retrieval, 4 due to inappropriate study design (no adult population/preclinical studies/no GBM), 2 for lack of outcome of interest (OS/PFS), 6 for lack of quantitative analysis of the outcomes. 21 articles were included in the review, including 2198 patients. Table 1 summarizes the characteristics of the included studies and their quality assessment, while clinical outcomes are detailed in Table 2.

**Table 1:** Overview of the baseline characteristics of the 21 included studies: Age, sex distribution, molecular targets, neurological symptoms, median Karnofsky score at onset, therapeutic strategy and follow up scheme

Author and year of publication	Patient sample (n)	Males / Females ratio	Mean age (years)	Target of the study	Median Karnofsky score at onset	Therapeutic strategy	Follow-up scheme
*ECM degradation molecules*							
Flannery et al. 2006	88		54,35	Cathepsin S (CatS) expression	70	RT (Gy) 0: 6,1% < 30: 9,8% 30–40: 35.35% 45: 3,65% 55: 17.05% 60: 100%	
Yang et al., 2013	206	122/84	53.6 (14–78)	CD147 expression	*CD147 high* = KPS ≥ 80 = 67 (59%) KPS < 80 = 6 (41%) *CD147 low* = KPS ≥ 80 = 72 (77%) KPS < 80 = 21 (23%)	*CD147 high expression:* GTR = 96 (85%) STR = (15%) *CD147 low expression*: GTR = n 79 (85%) STR = n 14 (15%)	Median follow-up 12.3 months (1–60)
*Cytoskeletal reorganization molecules*							
Aoki et al., 2007	136		56 (± 13)	Phosphorylated Pak1 levels in the cytoplasm and nucleus			
Garcia et al., 2012	59 (grade IV glioma, *n* = 49)	33/17	65 (34–76)	VAV1 expression		VAV 1 weak expression (0–1%) GTR = 18 (67%) VAV 1 intensity (5 or > 5%) = GTR = 18 (78%)	
*Epithelial to mesenchymal transition (EMT)*							
Li et al., 2020	52	116/71	37 (2–75)7	Twist expression *Twist low expression* = 27 *Twist high expression* = 25	KPS < 70 *Twist low expression* 10 (37%) *Twist high expression* 10 (40%)	High Twist expression GTR = 78% STR = 22% Low twist expression GTR = 67% STR = 33%	
Mikheev et al., 2015	27			Periostin expression			
Sun et al., 2020	21			NKCC1 expression			
Wang et al., 2013	38		45 (18–71)	Stromal periostin expression		GTR, adjuvant RT (60 Gy 2-cm margin and edema), adjuvant CTR	
*Vascular mechanisms*							
DeLay et al., 2012	21			Anti-VEGF therapy		GTR 31 days after BEV 9 received BEV monotherapy 12 received BEV + carboplatin, TMZ	Every 4–6 weeks MRIs with T1 post-contrast images and FLAIR
Jiguet-Jigliare et al., 2022	AVAglio trial: 577 (BEV, *n* = 283; placebo, *n* = 294)	181/102 (BEV) 187/107 (placebo)	57.0 (20–79) (BEV) 56.0 (21–79) (placebo)	Anti-VEGF therapy and MMP9 plasmatic levels correlation AVAglio trial	KPS 50–80 BEV 84 (30%) Placebo 84 (29%) KPS 90–100 BEV 199 (70%) Placebo 210 (71%)	GTR = BEV 119 (42%) Placebo 158 (54%) Adjuvant RT and TMZ + BEV or placebo every 2 weeks In the maintenance phase TMZ + BEV or placebo. In the monotherapy phase, BEV every 3 weeks until disease progressed, or toxic effects developed	MRI, clinical assessment, glucocorticoid use, plasma samples
Schaub et al., 2018	142 94 BEV/IRI vs 48 TMZ	68/26 BEV/IRI; 31/17 TMZ	56 (25–78)	Anti-VEGF therapy Effect on growth and migration GLARIUS trial	90 (70–100)	GTR = BEV/IRI 58 (50%) TMZ 24 (45%) STR = BEV/IRI 58 (50%) TMZ 29 (55%)	MRI T1 pre-contrast, FLAIR, T1 post-contrast,—minimum 1.5 Tesla Tumor invasiveness patterns were categorized into *diffuse* = signal on FLAIR extends diffusely at least 2 cm beyond the contrast enhanced area *non-diffuse*
Scribner et al., 2014	23 GBM at first recurrence necrosis ( +) n 11 (48%) necrosis (-) n 12 (52%)	Necrosis ( +): 8/3 Necrosis (-): 10/2	Necrosis ( +): 48.4 (27–73) Necrosis (-): 54.7 (29–65)	Anti-VEGF therapy Effect on growth and migration		RT + TMZ	MRI showing maximal effects of BEV causing reduction in contrast enhancement and FLAIR signal abnormality
Onguru et al., 2008	54	41/13	53.2 (20–74)	Cox-2 expression and MVD *Cox-2 IHC score* none = 6 low (1–3) = 35 moderate (4–6) = 11 high (7–9) = 2 *MVD* > *70* Bizarre vascular pattern = 36 Classic vascular pattern = 18			
Stadlbauer et al., 2018	52 with IDH1 wild type GBM	32/20	63.5 ± 12.6 (36–86)	Oxygen metabolism on BOLD MRI and VAM	KPS 60–65: 9 (17%) KPS 70: 3 (6%) KPS ≥ 80: 40 (77%)	EOR defined by a postoperative MRI within 48 h: GTR > 95%: presence of enhancing tumor STR ≤ 95%: absence of enhancing tumor	The MRI protocol included: FLAIR DW-EPI GRAPPA T1-weighted gradient-echo qBOLD + VAM using perfusion with dual contrast agents
Qiu et al., 2015	61	34/27	55.0 (18–78)	Neovascularization via Notch ligand, Delta-like ligand 4 (DLL4) and Jagged 1 (JAG1) expression	KPS ≥ 70: 23 (38%) KPS < 70: 38 (62%)	GTR + Adjuvant chemotherapy and radiotherapy: Radiotherapy = to limited fields Chemotherapy with TMZ for at least 4 cycles	3-month intervals in the first year; 6-month intervals thereafter Median follow up time: 56.2 weeks (20–144)
*RNA sequencing data*							
Qian et al., 2021	388	147/241	42 ± 12 years (8–79)	Annexin A1 (ANXA1) expression *ANXA1 high expression* = 296 (76%) *ANXA1 low expression* = 92 (24%)			
Zinn et al., 2012	78			Volume-Age-KPS (VAK) Volume-Age-KPS-MGMT (VAKM) classification			
*Apoptotic regulation mechanisms*							
Qian et al., 2017	91	46/45	49.38 ± 15.87 (13–85)	FoxO3a expression			
Stark et al., 2003	27	17/10	56 (29–73)	mdm2, EGFR and msh2 expression Recurrent GBM		GTR of initial tumor. Re-craniotomy for second tumor recurrence 7 (26%) RT at 154 Gy CTR 17 (63%): cisplatin/tamoxifen, gliadel, TMZ	Clinical examination and MRI studies every 3 months or in clinically suspected tumor recurrence
Stoyanov et al., 2022	45			Diaph3 expression			
*Tissue of origin*							
Yang et al., 2015	82	56/26		MRI associated textures to identify tissue of origin		GTR or STR + adjuvant chemo/radiotherapy	T1 post contrast MRI T2 FLAIR MRI

ECM, extracellular matrix; KPS, Karnofsky Performance Score; COX-2, Cyclooxygenase-2; MVD, microvessel density; ANXA-1, annexin 1; FOXO3a, Forkhead box O; epidermal growth factor receptor (EGFR); GBM, glioblastoma multiforme; CT, computer tomography; BOLD MRI, blood oxygen dependent level MRi; VAM, vascular architecture mapping; MGMT, promoter methylation status; GTR, gross total resection; STR, subtotal resection; EOR, extent of resection; MRI, magnetic resonance imaging; TMZ, temozolomide; BEV, bevacizumab; FLAIR, fluid attenuated inversion recovery; VAM, vascular architecture mapping; RT, radiotherapy; CTR, chemotherapy

**Table 2:** Overview of the outcomes of the 21 included studies: overall survival, progression free survival (PFS), author’s personal considerations

Author and year of publication	Molecular feature characterization technique	Overall survival (OS)	Progression free survival (PFS)	Author’s personal considerations
*ECM degradation molecules*				
Flannery et al. 2006	ELISA analysis (*n* = 41); Immunohistochemical analysis with anti-CatS antibody (*n* = 82); Kaplan–Meier survival analysis	*CatS concentrations* < *2.5 ng/mg* (*n* = 24) = 10 months *CatS concentration* > *2.5* *ng/mg* (*n* = 17) = 5 months *CatS score* < *30* (*n* = 60) = 10 months *CatS score* > *30* (*n* = 22) = 5 months		
Yang et al., 2013	Immunohistochemical analysis with Rabbit monoclonal CD147 primary antibody: *High CD147* = n 113 (54%) *Low CD147* = n 93 (45%) Kaplan–Meier survival analysis; Cox proportional hazards model;	High CD147 expression is associated with higher tumor grade *High CD147* = 10 months (median) *Low CD147* = 21 months (median)		High CD147 is associated to a 2.36 fold higher risk of death; CD147 was significantly correlated with patients’ KPS score < 80; CD147 expression was not found associated with patients’ sex, age at diagnosis, tumor size or extent of resection
Aoki et al., 2007	Immunohistochemical analysis with antibody specific to Pak1; phosphorylation on Thr; Kaplan–Meier survival analysis	*Ppak1 cytoplasmic levels*: C0 (*n* = 41), Median = 69 weeks C1 (*n* = 49), Median = 46 weeks C2 (*n* = 46), Median = 56 weeks *Ppak1 nuclear levels:* N1 (*n* = 23), Median 41 weeks N2 (*n* = 110), median 55 weeks		
Garcia et al., 2012	Immunohistochemical analysis; Comparative genomic hybridisation; Affymetrix U133A oligonucleotide microarrays	*VAV 1 weak expression* (0–1%) = 27 patients → OS 12 months *VAV 1 intensity* (5 or > 5%) = 23 patients → OS 10 months	VAV 1 weak expression (0–1%) = 6 months VAV 1 intensity (5 or > 5%) = 5 months VAV1-positive cells were located around GBM-triggered vasculature (18%) and surrounding tumour mass (20%)	Mesenchymal subtype: 9 Classic subtype: 2 Proneural: 1 Cytoskeletal dynamics were found associated to VAV1 expression
Li et al., 2020	Immunohistochemical analysis CD34 and PAS dual staining Kaplan–Meier survival analysis	*Low twist expression* = 62.63 months *High twist expression* = 14.23 months		*MVD* • Twist low expression = 29.53 ± 15.47 • Twist high expression = 34.62 ± 14.46 *VM* • Twist low expression = negative 94% • Twist high expression = negative 73%
Mikheev et al., 2015	Immunohistochemical analysis; Real-time qPCR; Total GBM = 27 High periostin = 12 (44%) Low periostin = 15 (55%)	*High periostin expression* = 15 months *Low periostin expression* = 56.4 months		
Sun et al., 2020	Immunohistochemical analysis NKCC1 0 intensity = 0 (0%) NKCC1 + 1 intensity = 2 (9%) NKCC1 + 2 intensity = 1 (5%) NKCC + 3 intensity = 18 (86%) Kaplan–Meier survival analysis; Gene expression profiling interactive analysis (GEPIA)	NKCC1 expression is associated with higher tumor grade *High NKCC1 expression* = mean survival percentage at 60 months 0% *Low NKCC1 expression* = mean survival percentage at 60 months 18%		NKCC1-mediated EMT may be related to loss of polarity, tight junctions and adhesion of epithelial cells, resulting in cell infiltration and migration → thus changes in the characteristics and morphology of cells
Wang et al., 2013	RNA extraction and microarray analysis; Immunohistochemical analysis *High periostin* = n 32 (84%) *Low periostin* = n 6 (16%); Kaplan–Meier survival analysis; Cox proportional hazard regression	*High periostin* = 459 days (median) *Low periostin* = 1098 (median)	*High periostin* = 382 days (median) *Low periostin* = 683 days (median)	GTR had no correlation with OS and recurrences (typically at the surgical margin) Periostin correlates to cell migration, cell proliferation, cell motility and extracellular matrix organization. It is correlated to high expression of MMP-9
DeLay et al., 2012	Molecular characterisation: Microarray gene expression analysis Immunohistochemical analysis; Radiographic volumetric analysis during BEV treatment; Real time PCR; Kaplan–Meier survival analysis Radiological classification based on FLAIR enhancement: *Non-enhancing BRG (NBGR)* = 9 *Enhancing BRG (EBGR)* = 12	*EBRG* = Median 79 weeks *NBRG* = Median 93 weeks		Distinct transcriptional phenotypes are associated to each subtype. *NBGR* is associated with increased expression of EMT markers: α5β1 integrin, N-cadherin, TWIST1, Pseudopodia. *EBGR* is associated with increased aquaporin 4 expression
Jiguet-Jigliare et al., 2022	Enzyme-linked immunosorbent assay; Immunohistochemical and immunofluorescence analysis; Magnetic sorting; Flow cytometry analysis; Real-time qPCR	*Low MMP9 levels* = median 18.8 months *High MMP9 levels* = median 13.6 months	Probability of PFS at 15 months *Low MMP9 levels* = 40% in Bevacizumab treated 20% in Bevacizumab untreated *High MMP9 levels* = 20% in Bevacizumab treated 30% in Bevacizumab untreated	MMP9 plasma levels decrease after GBM resection. No correlation between MMP9 activity and FLAIR volume, contrast enhancement volume or infiltrative pattern was found
Schaub et al., 2018	MRI scans; Wilcoxon test and Fisher’s test; Kaplan–Meier survival analysis *Growth patterns at baseline* = BEV/IRI Local 77 (82%) Multifocal 17 (18%) TMZ Local 38 (79%) Multifocal 10 (21%) *Invasiveness patterns at baseline* = BEV/IRI Non-diffuse 39 (41%) Diffuse 55 (59%) TMZ Non-diffuse 22 (46%) Diffuse 26 (54%) *Growth patterns at recurrence* = BEV/IRI Local 59 (63%) Multifocal 19 (20%) Distant 16 (17%) TMZ Local 30 (63%) Multifocal 12 (25%) Distant 6 (13%) *Invasiveness patterns at recurrence* = BEV/IRI Non-diffuse 27 (29%) Diffuse 67 (71%) TMZ Non-diffuse 18 (38%) Diffuse 30 (63%)	*Local growth pattern:* 17.8 months in TMZ vs 16.9 months in BEV/IRI *Multifocal growth pattern:* 13.0 months in TMZ vs 15.3 months in BEV/IRI *Distant growth pattern:* 17.3 months in TMZ vs 16.5 months in BEV/IRI *Diffuse growth pattern:* 15.3 months in TMZ vs 16.5 months in BEV/IRI *Non-diffuse growth pattern:* 17.8 months in TMZ vs 16.5 months in BEV/IRI *Change from non-diffuse* *to diffuse growth pattern:* 13.8 months in TMZ vs 15.5 months in BEV/IRI *Whole subcohort:* TMZ: 17.1 months BEV/IRI: 16.5 months	*Local growth pattern:* 6.1 months in TMZ vs 9.6 months in BEV/IRI treated patients *Multifocal growth pattern:* 2.6 months in TMZ vs 9.8 months in BEV/IRI treated patients *Distant growth pattern:* 8.2 months in TMZ vs 8.1 months in BEV/IRI treated patients *Diffuse growth pattern:* 3.1 months in TMZ vs 9.8 months in BEV/IRI treated patients *Non-diffuse growth pattern:* 6.1 months in TMZ 8.4 months in BEV/IRI *Change from non-diffuse to diffuse growth pattern*: 2.4 months in TMZ 8.2 months in BEV/IRI Whole subcohort: TMZ: 6.1 months BEV/IRI: 9.6 months	Despite likewise prolonged progression-free survival (PFS), there was no relevant difference in the overall survival (OS) between the treatment arms Some studies report that patients treated with BEV/IRI showed a tendency toward distant and diffuse progression. In this study no statistically significant data support this hypothesis
Scribner et al., 2014	MRI scans; Kaplan–Meier survival analysis		*Necrosis (-)* = 333 days *Necrosis (* +*)* = 178 days	GBM continues to grow even in the absence of angiogenesis by expanding necrosis and FLAIR. This is perpetuated by by active transport, a mechanism of hypoxia-driven brain invasion
Onguru et al., 2008	Immunohistochemical analysis with antibodies against Cox-2 and CD34; Algorithm for the standardized assessment of vascular patterns described by Preusser et al	*Bizarre vascular pattern* median 9 months *Classic vascular pattern* median 6 months (not statistically significant) COX-2 expression in classical vascular pattern was higher than bizarre pattern: IHC 3 vs. 2		Not statistically significant results on OS. Cox-2 is heterogeneously expressed in GBM without a significant association with MVD. However, the difference in Cox-2 expression between the classical and bizarre vascular pattern in glioblastoma cases was statistically significant
Stadlbauer et al., 2018	MRI scans; Kaplan–Meier survival analysis; Cox proportional hazards models	Median 224 days *	Median 173 days** *Glycolic dominated phenotype with functional neovasculature*: PFS percentage at 400 days = 22% *Necrotic/hypoxic dominated phenotype with approximately 50% of defective neovasculature*: PFS percentage at 400 days = 18%	*Dysfunctional neovasculature* = hypoxia with defective tumor neovasculature *Functional neovasculature* = hypoxia with functional tumor neovasculature (mitochondrial oxidative phosphorylation) *No neovascularization* = necrosis with highly defective vasculature *Neovascularization* = normoxic tumor with functional tumor neovascularization
Qiu et al., 2015	Immunohistochemical analysis for DLL4/JAG1 in tumor cells expression *DLL4 low* = < 10% of cells positive staining *DLL4 high* = ≥ 10% of cells positive staining *JAG1 low* = < 40% *JAG1 high* = ≥ 40% 5 microvascular patterns of formation identified: 1) *microvascular sprouting* (MS)—subtle capillary like microvessel; 2) *vascular cluster* (VC)—≥ 3 vessels aggregated without connective strom; 3) *vascular garland* (VG)—vessels clustered in garland-like formations with or without connective stroma; 4) *glomeruloid vascular proliferation* (GVP) ≥ 3 vessels ensheathed with connective stroma; 5) *vasculogenic mimicry* (VM)—hollow channels or networks with CD34-negative and acid-Schiff-positive staining *Type I MVP: MS, VC* *Type II MVP: VG, GVP, VM* MVD was defined as the number of manually counted vessels per square millimeter and presented as the mean of 5 hotspots; Kaplan–Meier survival analysis	*High JAG1* = 20% survival probability at 60 months *Low JAG1* = 50% survival probability at 60 months *High DLL4* = 20% survival probability at 60 months *Low DLL4* = 50% survival probability at 60 months *MVP type I* = 40% survival probability at 60 months *MVP type II* = 10% survival probability at 60 months		Type I MVP = 43 (70%) Type II MVP = 18 (30%) Mean MVD = 71 vessels/mm Type I MVP = 48.3 MVD, away from necrosis Type II MVP = 102.7 MVD, close to necrosis MVP type II consist of more pericytes compared to MVP type I JAG1 is vital in recruitment of vascular mural cells DLL4 is inversely related to MVD whereas JAG1 is positively correlated with MVD. DLL4 and JAG1 have opposing roles in regulating tumor angiogenesis
Qian et al., 2021	Single-cell RNA sequencing; Immunohistochemical analysis with antibodies against ANXA1; Gene set enrichment analysis; Kaplan–Meier survival analysis; Cox proportional hazard model analysis	Higher ANXA1 levels are associated with IDH wild-type and non-MGMT methylated phenotypes OS at 3000 days: *Low ANXA1* = 52% *High ANXA1* = 2%		*Low ANXA1:* more likely belong to proneural (PN) and neural (NE) subtypes and have a good prognosis *High ANXA1:* are more likely belong to mesenchymal (MES) and classical (CL) subtypes and have poor survival. Often associated to the following mutations: TP53, EGFR ANXA1 is involved in immune-related functions (expression of M2 macrophages) and inhibition of the glioma immune microenvironment
Zinn et al., 2012	MRI scans for preoperative tumor volumetry; Genomic analysis Ingenuity Pathway Transcription Factor analysis; Kaplan–Meier survival analysis; Cox proportional hazards likelihood ratio; 3-point VAK classification system: - Volume ≥ 30,000mm3 or ≥ 40 mm diameter = 1 point - Age ≥ 60 years = 1 point - KPS < 100 = 1 point *VAK-A* (good prognosis) = 0–1 points *VAK-B* (poor prognosis) = 2–3 points	Single factors *Total tumor volume* < *30.000mm3* = 18.5 vs 12 *Low T2/FLAIR volume* = 15.5 14 vs months *Age* < *60* = 18 vs 13 months *KPS* < *100* = 13.5 vs 20 months VAK classification *VAK-A* = 20 months *VAK-B* = 12 months		
Qian et al., 2017	Western blot; Immunohistochemical analysis with antibody against FoxO3a; *Low FoxO3a* = 26 (29%) *Moderate FoxO3a* = 25 (27%) *High FoxO3a* = 40 (44%) Kaplan–Meier survival analysis	*Low nuclear FoxO3a:* median OS at 13 months (*n* = 32) *High nuclear FoxO3a* median OS 8 months (*n* = 51)		Is critical in regulating cell autophagy activation via c-Myc, LC3B and Beclin
Stark et al., 2003	Immunohistochemical analysis with mAb anti-p53 (DO-1), anti-mdm2 (IF-2), anti-EGFR (H11) and anti-msh2 (AB-1); 0 = no staining; 1 = detectable staining < 5% of cells; 2 = nuclear staining 5–60%of the cells; 3 = > 60% staining of cells; p53 and mdm2 score of 0.1 = negative, 2.3 = positive Msh2 score of 0 ± 2 = reduced expression 3 = normal expression EGFR staining negative: up to 50% of cells stained. Positive: > 50% of cells stained; Wilcoxon test Log-rank test (Cox-Mantel)	No correlation observed between immunohistochemical expression indices and OS	No correlation observed between immunohistochemical expression indices and OS	Recurrent GBM was characterized by: *Reduced mdm2 and EGFR specimens ·* *Reduced msh2* *Reduced immunohistochemical scores for p53 and msh2* Recurrent GBM might represent a tumor stage of generally reduced protein expression caused by further dedifferentiation
Stoyanov et al., 2022	Immunohistochemical analysis with anti-Diaph3 antibodies; Diaph3 cutoff value for high vs low expression was 60%; High Diaph3 expression = 26 Low Diaph3 expression = 24; Kaplan–Meier survival analysis	*Low Diaph3:* 267.17 days *High Diaph3:* 246.19 days		No significant correlation observed between Diaph3 expression and patient survival or tumor size Diaph3 correlates with mTOR activity → Diaph3 can predict response to rapamycin and taxanes
Yang et al., 2015	MRI scans; Image preprocessing and MRI texture features extraction; Random Forest algorithm; Receiver Operating Characteristic (ROC) curves; AUC values obtained from MRI-texture features for predicting different GBM molecular subtypes and 12-month survival status *Classical subtype* 21 (26%) *Mesenchymal subtype* 29 (35%) *Neural subtype* 12 (15%) *Proneural subtype* 20 (24%)	Survival percentage at 12 months: *Classical subtype* 72% for classical subtype (T1 contrast MRI) *Mesenchymal subtype* 70% (T2 FLAIR MRI) *Neural subtype* 75% (T2 FLAIR MRI) *Proneural subtype* 82% (T1 contrast MRI)		

MVD, microvascular density; VM, vasculogenic mimicry; PCR, polymerase chain reaction; BRG, bevacizumab resistant glioblastoma; IHC, immunohistochemistry; GBM, glioblastoma multiforme; OS, overall survival; GTR, gross total resection; MMP-9, matrix metalloproteinase-9; TMZ, temozolomide, TMZ/IRI, temozolomide+irinotecan

*25 patients (48%) still alive at last contact were censored

**8 patients (15%) without progression at last contact were censored

**Fig. 2:**
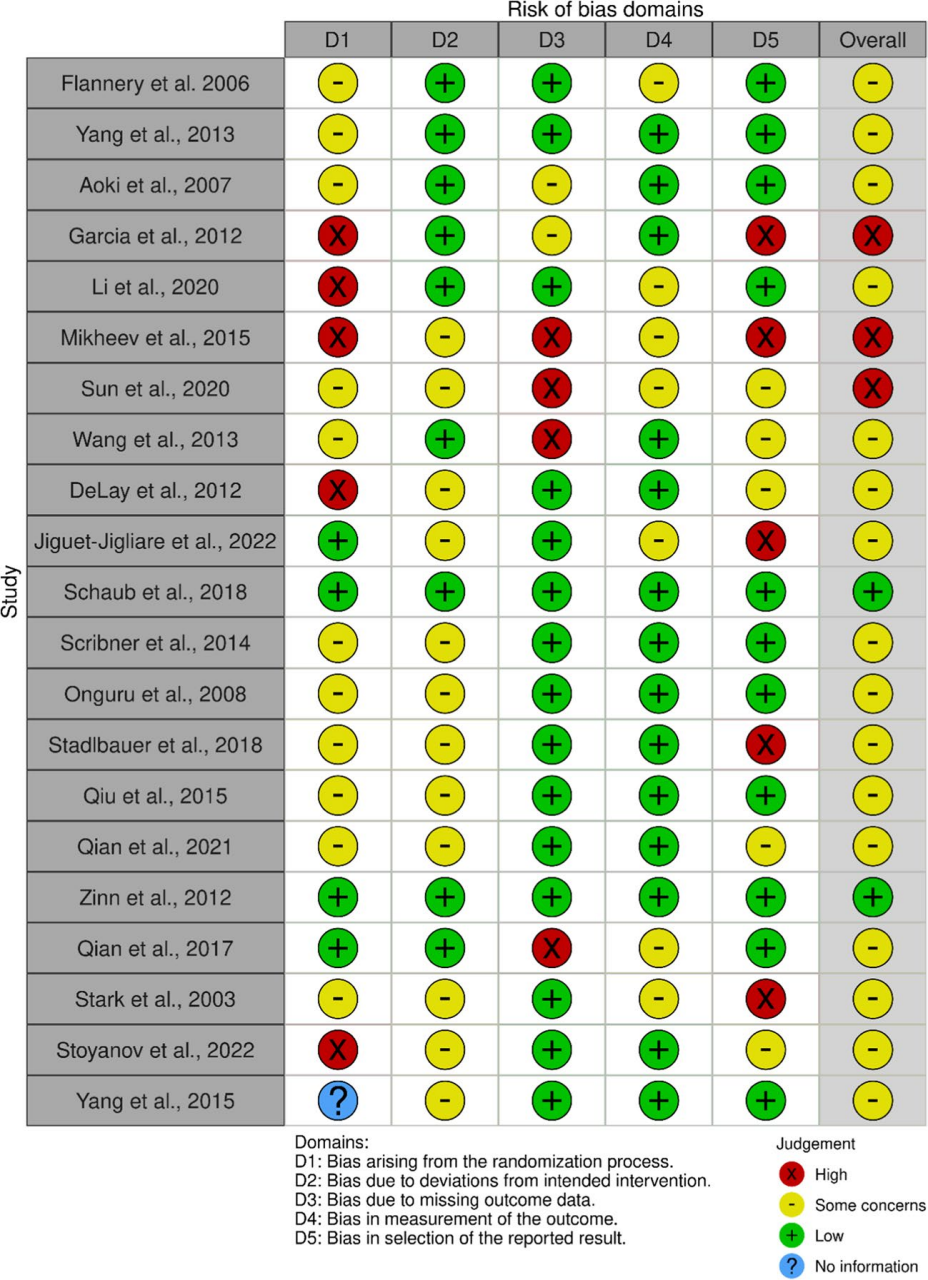
Robvis (visualization tool) for Risk of Bias of the 21 included studies

**Fig. 3:**
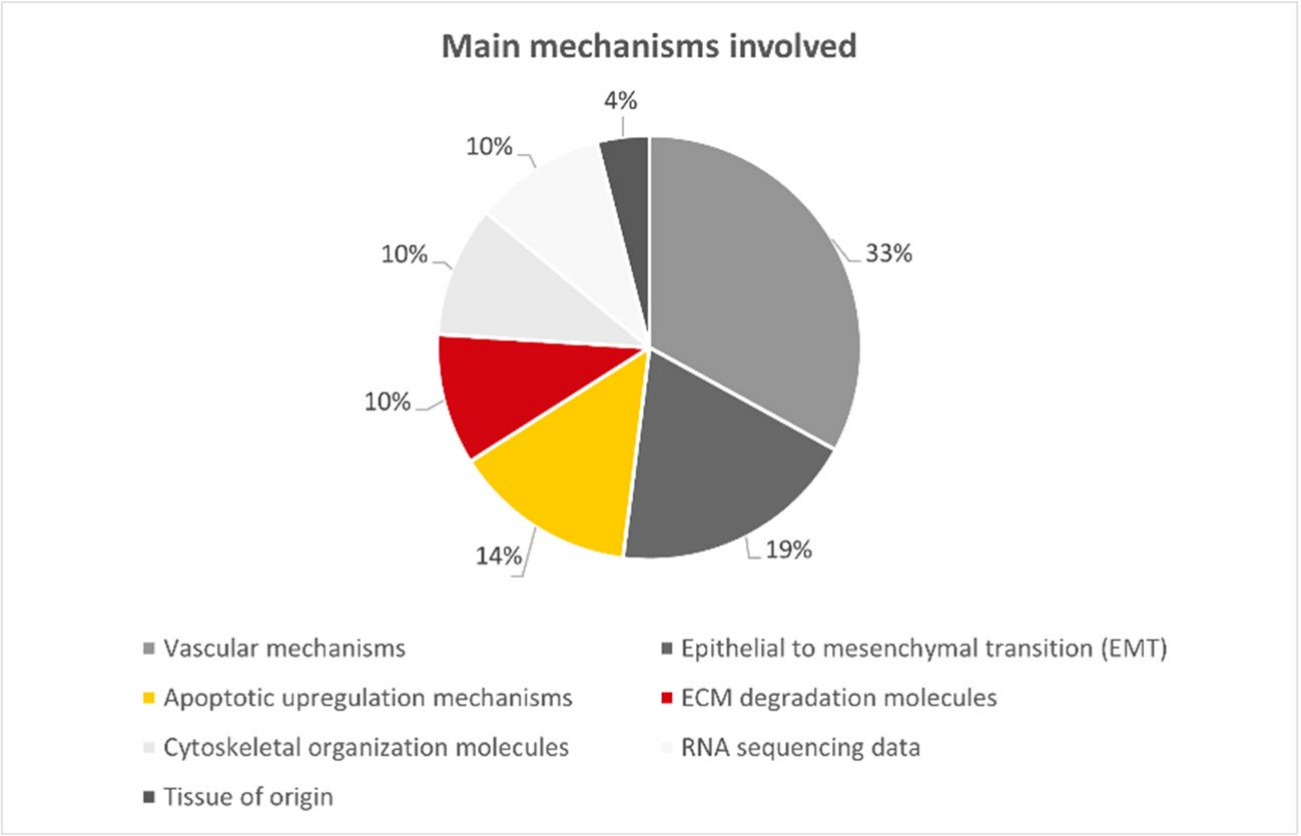
Pie Chart of the 21 study results according to the main categories

**Fig. 4:**
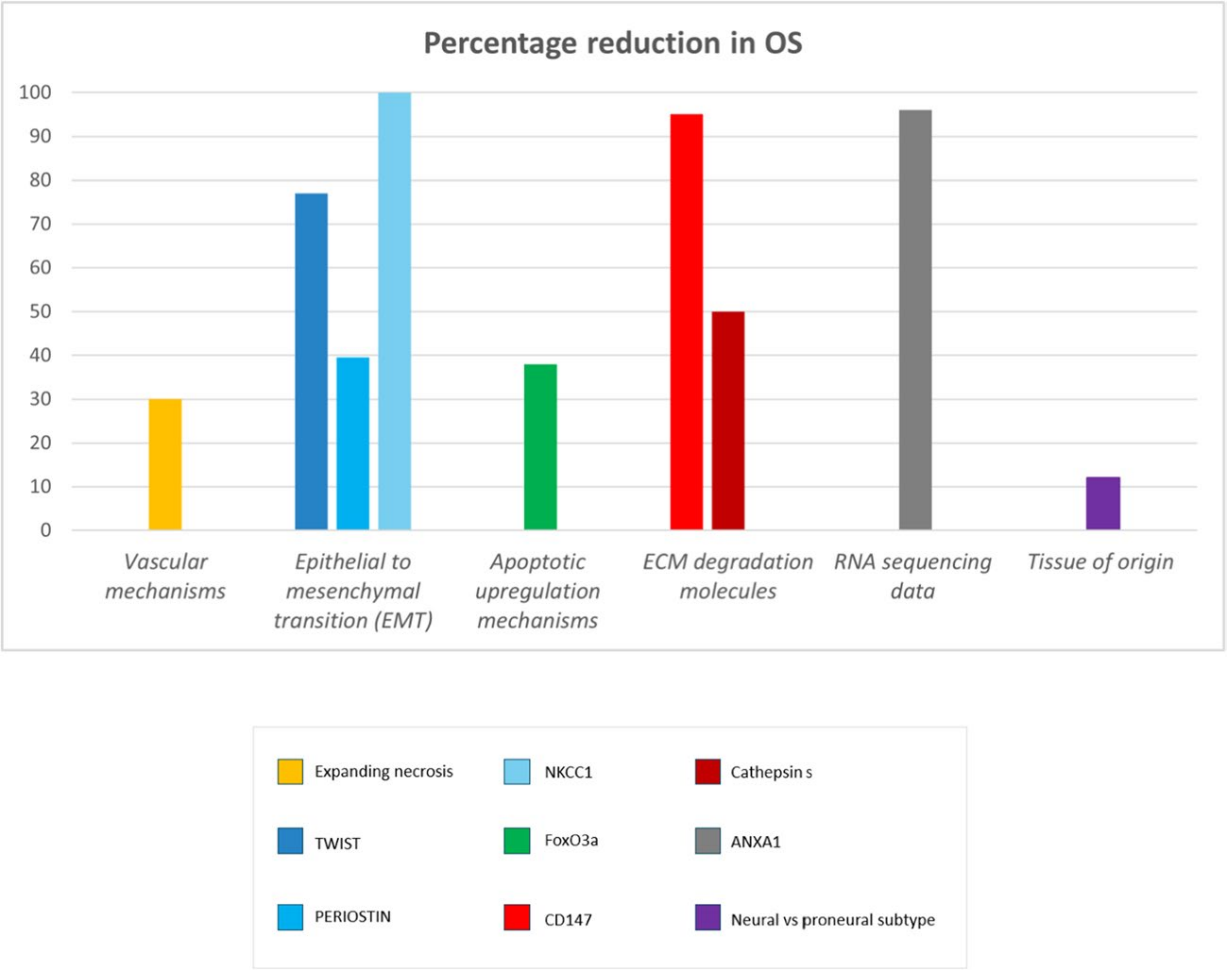
OS percentage reduction by mutational frames of the 21 study results

The included studies were classified in the following categories based on their main research interest: (1) *studies dealing with ECM degradation molecules* [16]*; studies dealing with cytoskeletal organization molecules* [17, 18]*; studies dealing with epithelial-to-mesenchymal transition* [19–22]*); studies dealing with vascular mechanisms *[23–29]*; studies dealing with RNA sequencing data* [30, 31]; *studies dealing with apoptotic regulation mechanisms* [32–34]*; and studies dealing with radiological patterns associated to a tissue of origin* [35]*.* Overall studies suffered from high variability and poor comparability, and studies were selected only for qualitative analysis [16] (Figs. 2, 3 and 4).

## Discussion

Our systematic review underscores the complex and multifaceted nature of glioblastoma invasion, highlighting significant findings from both laboratory and clinical perspectives. Molecular mechanisms conferring invasiveness and aggressiveness to central nervous system (CNS) tumours are becoming the current focus of patient’s clinical assessment [35], and our search organized them in the following macro-areas: *vascular mechanism, EMT, apoptotic regulation mechanism; ECM degradation molecules, cytoskeletal organization molecules; RNA sequencing data; tissue of origin.*

### Vascular mechanisms

The most common GBM patterns of invasion were vascular mechanisms, identified in 33% of the studies [23–28]. No statistically significant difference in OS was seen in bevacizumab treated GBM versus standard chemotherapy. Only a slightly increased PFS of 3.5 months was seen in bevacizumab + irinotecan therapy compared to standard temozolomide therapy alone [25]. However, 71% of the studies in this category dealt with bevacizumab therapy resistance [23–26, 29]. A common pattern of aggressive GBM behaviour was identified after bevacizumab resistance: expanding necrosis. Expanding necrosis is characterized by poorly vascularized tissue which appears as non-contrast enhancing, and hyperintense in FLAIR [23]. Its mechanisms of expansion are driven by hypoxia and are associated to a significant PFS percentage reduction, up to 47% [26]. At immunohistochemistry the deprivation-from-oxygen mechanism was proven by the substitution of aquaporin 4 by integrin α5β1 [23]. Other proposed mechanisms underneath bevacizumab resistance are the occurrence of EMT, supported by increased TWIST expression and appearance of pseudopodia resembling features [23]; and ECM involvement, supported by the expression of MMP9 during bevacizumab therapy, correlated to a median OS reduction of 5.2 months [24]. This highlights the importance to go beyond already explored therapeutic fields, among which bevacizumab therapy – whose controversial potential is supported by the occurrence of more aggressive behavioural patterns after treatment. This also enhances how molecular pathways are repetitively encountered in GBM behaviours, since EMT and ECM – which will be covered in the next paragraphs – are also involved in bevacizumab resistance.

Neoangiogenesis is also exploited by GBM to survive in poor oxygen conditions and is mainly regulated by cyclooxygenase-2 (Cox-2) enzyme expression. It can be characterized according to the microvascular density (MVD) of the newly formed vessels, as well as their vasculature architecture mapping (VAM) [28]. High MVD is paralleled by an increased incidence of necrotic areas and aggressive microvascular conformations (vascular garland, glomeruloid, and vasculogenic mimicry). These patterns were associated with an OS percentage reduction of 30% when compared to other microvascular behavioural patterns (microvascular sprouting and clustering) [29]. VAM can be studied on blood oxygen dependent level magnetic resonance angiography (BOLD-MRI) to identify hypoxia driven growth. Dysfunctional neovasculature – identified as hypoxia driven phenotype – was associated to lower PFS at 400 days when compared to functional oxygen rich vascularization [28].

This highlights the prognostic significance of MVD in stratifying patients into different risk categories; that BOLD-MRI could be a valuable diagnostic tool for identifying high-risk patients who are likely to benefit from treatments targeting hypoxic and neovascular regions; and that targeting the pathways involved in neoangiogenesis, such as through Cox-2 inhibitors, could potentially improve treatment efficacy and patient outcomes.

### Epithelial to mesenchymal transition (EMT)

EMT was identified as the second most common mechanism of GBM invasion, encountered in 19% of the studies [19–22]. Among them TWIST molecule is the most representative and associated to the potential of creating a vasculogenic mimicry (VM): which is the capacity to resemble endothelial cells enriched in ion channels. TWIST was associated to an OS percentage reduction of 77% as well as an increased microvascular density (MVD) [19].

Periostin, acting on stem cell niche, was associated with a mean OS reduction of 39.5 ± 20.5% [20, 22].

A potassium chloride co-transporter (NKCC1) – which induces the transformation of epithelial cells into mesenchymal cells via loss of polarity, tight junctions and adhesion molecules – was associated to an OS percentage reduction at 60 months of 100% [21].

This suggests that TWIST could serve as a valuable biomarker for identifying high-risk patients who may benefit from more aggressive treatment strategies and closer monitoring. Instead periostin has the potential utility to stratify patients based on their risk profile and tailor treatments that target the stem cell niches. The stark prognostic significance of NKCC1 suggests its potential as a target for novel therapeutic interventions aimed at inhibiting EMT processes.

### Apoptotic upregulation mechanisms

14% of the studies dealt with apoptotic upregulation mechanisms [32–34]. Two of them failed to establish correlations with MDM2, epidermal growth factor receptor (EGFR), MSH and Diaph3 expression and OS, highlighting the complexity of apoptotic regulation in GBM [33]. However, the identification of FoxO3a as a key controller of cell survival under hypoxic conditions presents a potential avenue for patient stratification and therapeutic targeting. FoxO3a expression was associated with a significant 38% reduction in OS, underscoring its prognostic value in identifying high-risk patients [32]. The multifaceted nature of apoptotic regulation necessitates a deeper understanding of the underlying mechanisms and their interplay with other pathways involved in GBM progression.

### ECM degradation molecule

10% of the studies dealt with ECM degradation molecules [16, 35], identifying CD147, a MMP, as being dramatically associated to OS reduction, with a percentage reduction of 95% [35]. Cathepsin S, a lysosomal proteinase capable of degrading the ECM molecules laminin, collagen, and elastin, was associated to a less dramatic OS percentage reduction of 50% [16]. There was no difference in the surgical approach of subtotal resection (STR) versus gross total resection (GTR) in different CD147 expression groups. A percentage of Karnofsky performance score (KPS) < 80 was higher in the high CD147 group (41%), compared to the low CD147 group (23%) [35]. According to these data, the expression of CD147 is associated to a significant reduction not only in OS, but also in the quality of life (qOL), showing significantly lower KPSs in the mutated group. This highlights the importance of implementing this marker in the diagnosis to assess, beyond the need for more aggressive therapeutic strategies, personalized supportive plans such as rehabilitation structures due to the significant reduction in qOL.

### Cytoskeletal organization molecules

Cytoskeletal organization molecules, such as P21-activated kinase 1, whose mechanism of invasion consist in the rearrangement of cytoskeletal actin filaments, and VAV1, responsible for microtubular rearrangement, were identified in 10% of the studies. None of which was found associated to a statistically significant reduction in OS. Also PFS doesn’t associate to a significant difference between the two groups [17, 18].

### RNA sequencing data

10% of the studies dealt with RNA sequencing data [30, 31], which allowed to classify GBM according to the tissue of origin. Proneural (PN) and neural (NE) subtypes were identified as better prognostic compared to mesenchymal (MES) and classical subtypes (CL). The latter two are associated to high ANXA1 expression and have an OS percentage reduction of 96%. This dramatic OS reduction is thought to be associated to an immune downregulation induced by ANXA1, favouring the inhibitory glioma microenvironment [30]. This stresses the importance to implement the role of the immune system in the determination of glioma microenvironment [36]. Zinn et al. proposes an innovative classification of OS according to tumor volume, patient age and KPS. The group VAK-A, associated to lower age, higher KPS and lower tumor volume, was associated to a higher OS compared to VAK-B (20 months vs 12 months). In addition, MGMT promoter methylation status resulted in a 10.5-month additional survival benefit for VAK-A group [31].

### Tissue of origin

1 study identified a correlation between MRI (T1 contrast, T2 FLAIR) features and tumoral tissue of origin [37]. Different survival percentages at 12 months have been associated to different progenitor tissues: classical subtype 72%, mesenchymal subtype 70%, neural subtype 75%, proneural subtype 82% [37]. This highlights how radiological patterns can already be implemented in GBM risk stratification.

### Clinical relevance

The molecular patterns highlight the reason why it is almost impossible to achieve a complete surgical resection in GBM and its propensity for local recurrence. Our review unveils the need for integrated therapeutic approaches that not only target the tumour cells, but also modify the tumour microenvironment and address specific invasion pathways [36, 38]. From the data retrieved, current therapeutic strategies are facing important challenges in terms of concrete improvement in OS, such as in the case of bevacizumab [23–26, 29]. In addition, only 1 study reported an already established clinical correlation of a molecular target: Diaph3 is associated with a better response to chemotherapy with rapamycin and taxanes [34]. This highlights how far we are from efficient therapeutic strategies in the direction of personalized medicine. Incorporating such molecular targets into the diagnostic and prognostic GBM workup could enhance patient stratification in this direction. By identifying patients with high expression levels of TWIST, NKCC1, and CD147 among others, clinicians can better predict tumor aggressiveness and hopefully tailor patients’ treatment. As a second pass, these markers could guide the development of targeted therapies ultimately improving patient outcomes. Overall, integrating molecular profiling into routine clinical assessment represents a promising approach to refining GBM management and enhancing survival rates, and preliminary data encourage that we’re looking in the right direction.

Overall, comparing molecular tumor profiles before and after therapy, as well as examining tissue from responders versus non-responders, is crucial for generating hypotheses to identify combination treatments that overcome resistance. This approach might also help discover new biomarkers to predict escape mechanisms in individual patients [39]. In this context, as suggested by Chen et al., may be useful to pursue more early-phase clinical trials where longitudinal biopsies of tissues and fluids are embedded in the science of the therapy under study, instead of the traditionally designed “window of opportunity” trials which – as mentioned before – have failed to obtain significant results in glioma treatment [36].

## Limitations

Big heterogeneity exists between the studies included in this review, and some of them show contrasting data [30, 37]. Only studies on human subjects in vivo were included. However, the investigation of molecular patterns of invasion requires a thoughtful examination of cells behaviour in in-vitro conditions. No sufficient data reporting mean values and standard deviation (SD) for OS and PFS were retrieved for meta-analysis, limiting the statistical quality of information.

Furthermore, we acknowledge that our biggest limitation was the search limited to a single database (PubMed/Medline). While this database provides comprehensive coverage in the field of neuro-oncology, utilizing additional databases such as Embase or Scopus could have expanded the scope of our findings. This limitation may have resulted in the omission of relevant studies published in journals not indexed by PubMed. Future studies will aim to incorporate multiple databases to ensure a more exhaustive review of the literature. Another limitation in our search strategy was not include the term “high grade glioma”. This could have missed more recent studies using WHO 2021 nomenclature.

## Conclusions

Our systematic review has shed light on the intricate molecular mechanisms underlying invasiveness and aggressiveness in GBM. Through a comprehensive analysis, we have synthesized evidence that not only elucidates the pathways driving tumor progression but also highlights their implications for patients’ prognosis and treatment strategies. The insights garnered from this review underscore the importance of integrating molecular profiling into clinical assessments to refine prognostic predictions and tailor therapeutic interventions effectively. Moving forward, leveraging this knowledge holds the potential to revolutionize patient care by enabling personalized therapeutic approaches that target the specific molecular aberrations driving tumor invasiveness and aggressiveness. Further research endeavours are warranted to deepen our understanding of these mechanisms and translate these findings into tangible clinical benefits.

## Data Availability

The data supporting the findings of this study are available within the article and its supplementary materials. Any additional data can be made available upon reasonable request. Please contact the corresponding author, Veronica Percuoco, at percuocoveronica@gmail.com for inquiries regarding data access.
